# Investigation of genotype diversity of 7,804 norovirus sequences in humans and animals of China

**DOI:** 10.1515/biol-2022-0511

**Published:** 2022-11-07

**Authors:** Manyu Li, Kejian Li, Haiyun Lan, Xiaotian Hao, Yan Liu, Cheng Zhou

**Affiliations:** Division I of In Vitro Diagnostics for Infectious Diseases, Institute for In Vitro Diagnostics Control, National Institutes for Food and Drug Control, 2 Tiantanxili Rd, Dongcheng District, Beijing 100050, China; Division I of In Vitro Diagnostics for Infectious Diseases, Institute for In Vitro Diagnostics Control, National Institutes for Food and Drug Control, Dongcheng District, Beijing 100050, China

**Keywords:** genotype diversity, norovirus, zoonosis

## Abstract

Norovirus is a prominent enteric virus responsible for severe acute gastroenteritis disease burden worldwide. In our current study, we analyzed 7,804 norovirus sequences of human and animals in China which were detected from 1980 to 2020 from GenBank. The GenBank database was searched up to May 2021 with the following search terms: “norovirus” or “norwalk virus” and “China.” The 7,804 norovirus sequences were collected and evaluated by phylogenetic analysis using MEGA X software package. The online typing tool (https://www.rivm.nl/mpf/typingtool/norovirus/) was used to confirm the genotypes. There were 36 norovirus genotypes prevailing in China. GII.4 was the most prevalent genotype, and GII.2, GII.3 and GII.17 also emerged during different time periods. Most sequences were detected in East China (41.72%, 3,256/7,804), but different norovirus genotypes were distributed widely across the country. A variety of norovirus genotypes, including GI, GII, GIII, GIV, GV, GVI, GVII and GX, were reported in different animals. Furthermore, a GI.3 sequence detected from animal had high identity with norovirus detected in human from the same region, indicating the potential norovirus zoonotic transmission in China. In conclusion, these results indicated that norovirus sequences with considerable genetic diversity distributed widely in China, with potential reverse zoonotic transmission from human to animals.

## 1 Introduction

Norovirus is a leading cause of epidemic and sporadic nonbacterial gastroenteritis of all age groups worldwide, with an estimated 684 million cases annually resulting in 212,000 deaths [1–3]. Its infection can be serious in young children, the elderly and immunocompromised individuals [4]. Norovirus is a single-stranded, positive sense RNA virus, which belongs to the Caliciviridae family [5]. Currently, it can be divided into at least ten genogroups (GI–GX) and 48 genotypes, of which GI, GII, GIV, GVIII and GIX infect humans [6]. Despite its high degree of diversity, GII.4 is the predominant genotype detected in norovirus outbreaks worldwide [7]. But other genotypes have emerged in some regions of the world [8,9]. It is reported that norovirus infection accounted for 32.21% of all-age patients with acute diarrhea conducted in China, playing a significant role in the etiology of diarrhea [10]. Therefore, it is essential to understand the genotype diversity, dominant variants and variant replacement patterns of norovirus in China, which is valuable for taking preventive measures.

Animal noroviruses have been found in a wide range of hosts, including pigs, cattle, dogs, cats foxes, raccoon dogs, yaks and so on [11–14]. The interspecies transmission of noroviruses is not well understood and the detection of human-like norovirus genotypes in some farm animals, wild animals and pets, indicating the possible zoonotic transmission [15]. Therefore, the virus diversity, geographic distribution and probability of interspecies transmission of norovirus infections of animals in China need to be investigated.

Some studies have been done to investigate the phylogeography of norovirus in China [16–19]. However, there has been no detailed report about the overall genotype diversity of norovirus of both human and animals and the possibility of zoonosis transmission in China during the past 20 years. In this study, we retrieved 7,673 human norovirus sequences and 131 animal norovirus sequences detected in China from GenBank. By analyzing these sequences, we aimed to summarize the molecular epidemiology of noroviruses in China, the geographical and temporal distribution of the different genotypes across the country and the potential interspecies transmission of norovirus.

## 2 Materials and methods

### 2.1 Norovirus sequences search and retrieve strategy

The GenBank database was searched up to May 2021 with the following search terms: “norovirus” or “norwalk virus” and “China.” Sequences which contain the capsid region (ORF2) with isolation time and region were included. Other sequences were excluded. A total of 7,804 norovirus sequences including 7,673 human norovirus sequences and 131 animal norovirus sequences were retrieved. These sequences were detected in China from 1980 to 2020. Of all the 7,804 sequences, 166 sequences were complete genome sequences and 7,638 sequences were partial genome sequences (fragment sizes ranging from 218 to 7,238 bp). Of the 131 animal sequences, 71 were from cattle, 8 were from mouse, 2 were from rhesus monkey, 33 were from dog, 2 were from yak, 2 were from bat, 3 were from pig, 2 were from rat, 7 were from cat and 1 was from chimp.

The geographic regions were divided into East China, South China, North China, Southwest China, Northeast China, Central China and Northwest China. East China includes Shandong, Anhui, Jiangsu, Jiangxi, Zhejiang, Shanghai, Fujian and Taiwan; South China includes Guangdong, Guangxi, Hainan, Hong Kong and Macau; North China includes Beijing, Tianjin, Hebei, Shanxi and Inner Mongolia; Southwest China includes Chongqing, Sichuan, Guizhou, Yunnan and Tibet; Northeast China includes Liaoning, Heilongjiang and Jilin; Central China includes Henan, Hubei and Hunan; Northwest China includes Shaanxi, Gansu, Qinghai, Ningxia and Xinjiang.

### 2.2 Sequence comparison and phylogenetic analysis

The online typing tool (https://www.rivm.nl/mpf/typingtool/norovirus/) was used to confirm the genotypes [20]. Nucleotide sequences were aligned and analyzed by the MEGA X software package (version X, www.megasoftware.net). The alignment of all sequences was performed by Clustal X of MEGA X software and then amended manually. The compatible nucleotide substitution model (GTR + I + G) was determined by Modeltest v. 3.7 [21]. The phylogenetic tree of animal norovirus sequences was constructed based on the partial ORF2 region (218 bp). Reference sequences belonging to different genotypes were obtained from previous reference for comparison in this study (Table S1) [6]. The phylogenetic tree was constructed by the maximum likelihood method in MEGA X. One thousand bootstrap replicates were used to calculate the percentages of the branches obtained. Bootstrap values which were more than 70% were shown and were regarded as the evidence of a phylogenetic grouping. If more than two sequences which were published in the same study and classified into the same sub-genotypes, only one sequence was listed on the dendrogram in this study to make the data easier to recognize. After the lengths of the sequences were adjusted to the same, the nucleotide identity comparisons were performed by NCBI Blast website (https://blast.ncbi.nlm.nih.gov/Blast.cgi).

## 3 Results

### 3.1 Phylogenetic analysis of human and animal norovirus sequences from China

The results showed that human norovirus sequences from China were all classified into GI (405 sequences), GII (7,259 sequences), GIX (8 sequences) and GIV (1 sequence) (Table 1). Among these, human norovirus sequences which belonged to GI can be further divided into GI.1, GI.2, GI.3, GI.4, GI.5, GI.6, GI.7, GI.8 and GI.9. Human norovirus sequences which belonged to GII can be further classified into GII.1, GII.2, GII.3, GII.4, GII.5, GII.6, GII.7, GII.8, GII.12, GII.13, GII.14, GII.16, GII.17, GII.20 and GII.21. GIV belonged to GIV.1 and GIX belonged to GIX.1.

**Table 1:** Genotypes of human norovirus sequences from China

Genotype (number)		Number of sequences
GI (405)	GI.1	21
	GI.2	108
	GI.3	84
	GI.4	32
	GI.5	41
	GI.6	86
	GI.7	9
	GI.8	11
	GI.9	13
GII (7,259)	GII.1	20
	GII.2	820
	GII.3	821
	GII.4	3,067
	GII.5	10
	GII.6	212
	GII.7	18
	GII.8	34
	GII.12	143
	GII.13	290
	GII.14	24
	GII.16	1
	GII.17	1,364
	GII.20	2
	GII.21	433
GIV (1)	GIV.1	1
GIX (8)	GIX.1	8

**Figure 1:**
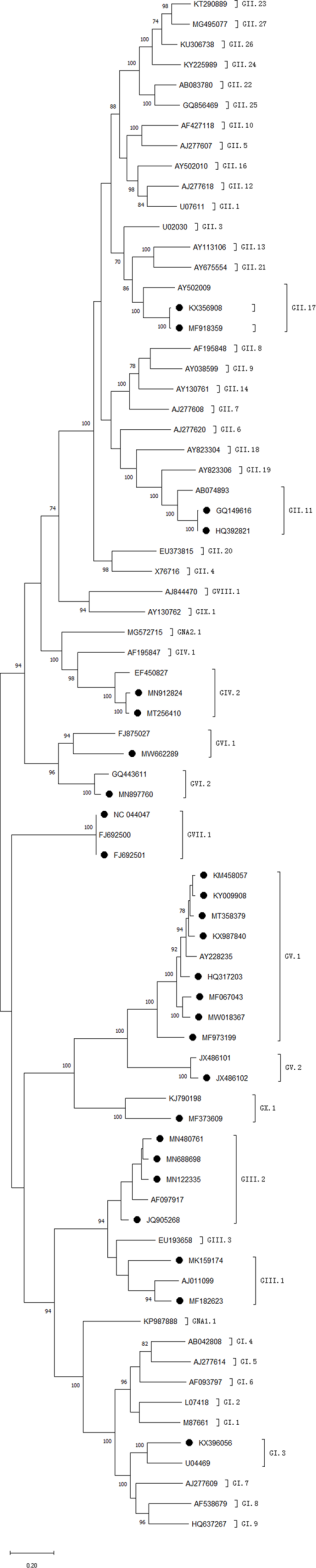
Phylogenetic trees representing animal norovirus genotypes. If more than two sequences which were published in the same study and classified into the same genotypes, only one sequence was listed on the dendrogram in this study. Bootstrap values which were less than 70% are not shown. The potential genotypic designations are shown outside of the square bars. ORF2 (capsid) regions of the sequences are compared and shown. ● represents the sequences from animals. The sequences without ● are the reference sequences.

Based on the phylogenetic tree constructed, we found that norovirus detected in animals of China belonged to GI (1 sequence), GII (5 sequences), GIII (73 sequences), GIV (9 sequences), GV (10 sequences), GVI (28 sequences), GVII (3 sequences) and GX (2 sequences) (Figure 1). Of these sequences, GI can be divided into GI.3, GII can be divided into GII.17 and GII.11, GIII can be classified into GIII.1 and GIII.2, GIV belonged to GIV.2, GV can be divided into GV.1 and GV.2 and GVI can be classified into GVI.1 and GVI.2. GVII and GX can be divided into GVII.1 and GX.1, respectively. Cattle norovirus mainly belonged to GIII.1 and GIII.2, and yak norovirus were all classified into GIII.2. Mouse norovirus all belonged to GV.1 and rat norovirus all belonged to GV.2. Dog norovirus can be classified into GIV.2, GVI.1, GVI.2 and GVII.1. Cat norovirus all belonged to GIV.2. Monkey norovirus were divided into GII.17 and chimp norovirus was GI.3. Pig norovirus belonged to GII.11and bat norovirus belonged to GX.1.

### 3.2 Geographic and temporal distribution of norovirus genotypes in China

Norovirus genotypes are distributed widely in China (Table 2). Among all the norovirus sequences, most sequences were detected in East China (43.31%, 3,380/7,804), followed by South China (24.83%, 1,938/7,804) and North China (20.89%, 1,630/7,804). Most of GI and GII sequences were also detected in East China. GIV sequence was detected in North and Southwest China. GIX.1 sequences were detected in East China, Southwest China, South China and Central China. GIII sequences were mostly detected in Southwest China. Most of GV sequences were detected in North China and most of GVI sequences were detected in Southwest China. Some genotypes of norovirus were mainly confined to certain areas, such as GX.1 was only reported in East and Southwest China.

**Table 2:** Norovirus genotypes geographic distribution in China

Genotype (number)	Region (percentage)						
	North China (%)	Northeast China (%)	East China (%)	Central China (%)	South China (%)	Southwest China (%)	Northwest China (%)
GI.1 (21)	—	—	71.43	—	28.57	—	—
GI.2 (108)	20.37	12.96	39.81	2.78	12.96	—	11.11
GI.3 (85)	34.12	—	43.53	2.35	18.82	1.18	—
GI.4 (32)	18.75	3.13	56.25	6.25	12.50	3.13	—
GI.5 (41)	2.44	—	80.49	—	14.63	2.44	—
GI.6 (86)	4.65	—	76.74	—	16.28	2.33	—
GI.7 (9)		—	88.89	—	11.11	—	—
GI.8 (11)	9.09	—	72.73	—	18.18	—	—
GI.9 (13)	38.46	—	46.15	—	15.38	—	—
GII.1 (20)	15.00	—	80.00	—	5.00	—	—
GII.2 (820)	11.34	0.37	35.37	18.29	23.78	8.17	3.05
GII.3 (821)	56.47	0.12	24.00	4.51	12.18	0.85	—
GII.4 (3,067)	24.36	1.96	38.93	1.27	31.92	2.87	0.65
GII.5 (10)	10.00	—	70.00	—	20.00	—	—
GII.6 (212)	12.26	—	58.96	6.13	12.26	4.25	6.13
GII.7 (18)	22.22	—	44.44	5.56	16.67	11.11	—
GII.8 (34)	2.94	—	11.76	—	85.29	—	—
GII.11 (3)	100	—	—	—	—	—	—
GII.12 (143)	77.62	—	14.69	4.20	2.10	1.40	—
GII.13 (290)	4.14	—	38.62	0.69	4.14	52.41	—
GII.14 (24)	8.33	—	62.50	8.33	12.50	8.33	—
GII.16 (1)	100	—	—		—	—	—
GII.17 (1,366)	2.12	1.76	55.86	5.93	36.02	0.07	—
GII.20 (2)	100	—	—	—	—	—	—
GII.21 (433)	9.24	—	86.14	—	4.62	—	—
GIII.1 (68)	—	—	—	—	—	100	—
GIII.2 (5)	20.00	—	—	20.00	—	40.00	20.00
GIV.1 (1)	100				—		
GIV.2 (9)	—	—	—	—	—	100	—
GV.1 (8)	50.00	—	37.50	—	12.50	—	—
GV.2 (2)	—	—	—	—	100	—	—
GVI.1 (2)	—	—	—	—	—	100	—
GVI.2 (26)	—	—	—	—	—	100	—
GVII.1 (3)					100		
GIX.1 (8)	37.50	—	—	25.00	12.50	25.00	
GX.1 (2)	—	—	50.00	—	—	50.00	—

**Figure 2:**
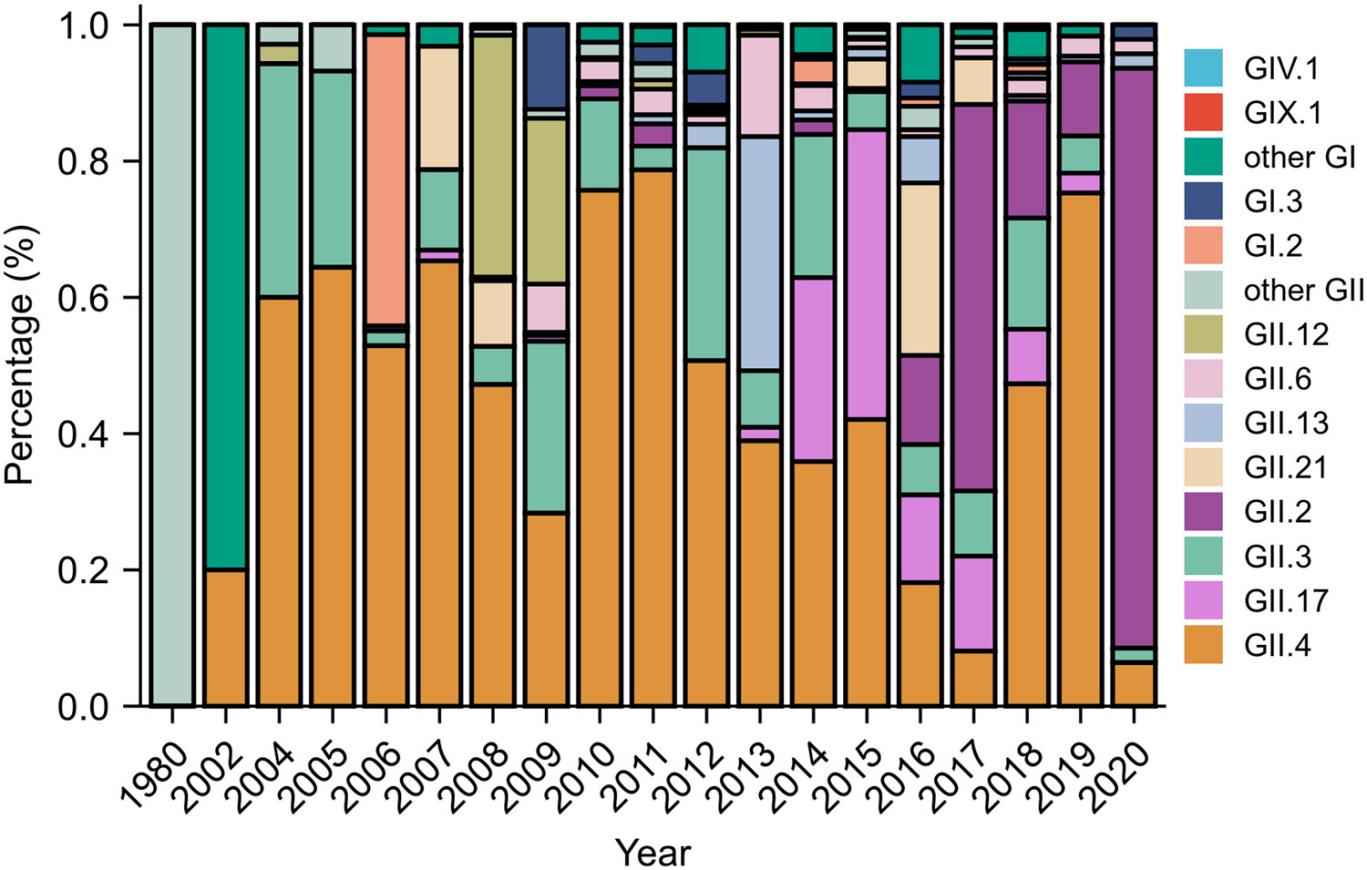
Temporal distribution of genotypes of human norovirus sequences in China. Different colors represent different genotypes of norovirus.

Figure 2, Tables S2 and S3 show the dynamic temporal change of human norovirus genotypes. As time progressed, the diversity of norovirus genotypes expanded. In 1980 and 2002, the most prevalent genotypes were GII.20 (100%) and GI.4 (80.00%), respectively. GII.4 was the most prevalent genotype between 2004 and 2014. In 2015, 2016 and 2017, GII.17 (42.53%), GII.21 (25.33%) and GII.2 (56.72%) were the most predominant genotypes, respectively. In 2018 and 2019, GII.4 genotype was the most prevalent genotype. In 2020, GII.2 was the most prevalent genotype (85.11%).

### 3.3 Potential norovirus zoonotic transmission in China

To investigate the potential zoonotic transmission of norovirus, we compared the identity of animal and human norovirus nucleotide sequences detected in the same geographical areas. The results showed that the chimp norovirus (KX396056) had the highest sequences/amino acid identity (84.7%) with the human GI.3 (KP753280), indicating the potential cross-species transmission between humans and animals in China.

## 4 Discussion

Norovirus outbreaks have been frequently reported in China and have caused a considerable disease burden [22]. Although there have been some studies about the phylogeography of norovirus in China [16–19], no study has summarized the overall genotype diversity of both human and animal norovirus and their potential zoonosis transmission in China during the past 20 years. Our study showed that the genotypes of norovirus in China included GI, GII, GIII, GIV, GV, GVI, GIX and GX. The percentages of GI, GII, GIII, GIV, GV, GVI, GIX and GX were 5.20% (406/7,804), 93.08% (7,264/7,804), 0.94% (73/7,804), 0.13% (10/7,804), 0.13% (10/7,804), 0.40% (31/7,804), 0.10% (8/7,804) and 0.03% (2/7,804), respectively, showing that GII was the most popular norovirus genotype in China. The results also showed that there were 36 genotypes throughout China. Among all genotypes, GII.4 (3,067/7,804, 39.30%), GII.17 (1,364/7,804, 17.48%), GII.2 (820/7,804, 10.51%) and GII.3 (821/7,804, 10.52%) were the predominant genotypes. This was in consistent with the global distribution of norovirus genotypes, whose predominant genotype is GII.4 and has been responsible for approximately 50% of outbreaks during the past several decades [23]. However, we found that GII.17, GII.2 and GII.3 also played an important role in the norovirus disease burden in China. Some studies predicted that these sequences may end the GII.4 era [24], but our study showed that GII.4 was still the most prevalent genotype and whether other genotypes can replace its predominance remains to be monitored.

In the present study, the results suggested that norovirus genotypes were detected in all seven regions of China, with most sequences detected in East China (43.31%, 3,380/7,804). This may be related with the local concerns, eating habits and geographic characteristics of East China. Among the predominant genotypes, most of GII.4 (38.93%), GII.2 (35.37%) and GII.17 (55.86%) were detected in East China and most of GII.3 (56.47%) were detected in North China, indicating that different predominant genotypes were circulating in different regions. This may be explained by the particularity of transmission of different genotypes and where the outbreaks occurred [25]. The temporal analysis of these human norovirus sequences suggested that the diversity of genotypes increased during the past 20 years. GII.4 has been particularly prevalent since 2002, and other genotypes (GII.17, GII.21 and GII.2) also became prevalent as time went by. The reason for the change of norovirus genotype among humans in China may be partly due to several outbreaks of different genotypes [26–29]. Although the sequences searched from GenBank in this study were affected by surveillance bias towards different regions, these results provided evidence that new norovirus genotypes continue to emerge and the differences in strain transmission remain unclear.

A variety of norovirus genotypes were obtained from animals in China, including cattle, mouse, rhesus monkey, dog, yak, bat, pig, rat, cat and chimp. GI and GII were mainly found in rhesus monkey, chimp and pig. GIII.1 and GIII.2 were all detected in cattle and yak. GIV was detected in dog and cat. GV.1 was detected in mouse and GV.2 was detected in rat. Both GVI.1 and GVI.2 were detected in dog. Besides, the norovirus sequences detected in bat all belonged to GX.1. We further aimed to investigate whether norovirus can jump the species barrier. The norovirus detected in chimps, which belonged to GI.3, showed high identity with human norovirus detected in the same regions, indicating the possibility of cross-species transmission. However, sequences detected in other animals, such as pigs, cattle, dogs and so on, did not show high identity with human norovirus in China. The norovirus sequence detected in chimps (GenBank No. KX396056) which belonged to GI.3 was detected from diarrheic chimps, indicating that human norovirus can cause symptoms in animals. Some phylogenetic analysis of noroviruses in animals showed that there may be an interspecies transmission of noroviruses [30]. To date, there is a lack of research on animal norovirus’s detection in human stool but it has been shown that human noroviruses could infect livestock animals such as pigs [31–34]. Our study indicated that human norovirus might be a reverse zoonosis pathogen, since the results showed that human noroviruses were detected in animals than the reverse. However, results in this study only indicated the zoonotic potential by comparing the identity of sequences and whether norovirus is a zoonotic or reverse zoonotic pathogen still remains to be further investigated.

In conclusion, human and animal noroviruses are widespread in China. This study has several limitations: (a) the sequences searched from GenBank were related with the local concern and number of studies, which cannot represent the whole temporal and geographical distribution of epidemic virology; (b) there is a possibility that there were missing sequences because their information did not contain the keywords. But this study still provided the genotype diversity of both human and animal noroviruses to some extent. The predominance of norovirus GII.4 is very clear, with a great diversity of genotypes circulating at a low frequency. Human norovirus might be a reverse zoonosis pathogen and the zoonosis still needs further investigation.

## Supplementary Material

Supplementary Table
