# Supplementary material for: Investigation of genotype diversity of 7,804 norovirus sequences in humans and animals of China
Source: Open Life Sci. 2022 Nov 7;17(1):1429–35. doi: 10.1515/biol-2022-0511 (PMC9644719; doi:10.1515/biol-2022-0511)
Supplement: Supplementary Table [file biol-2022-0511-sm.pdf]

Supplementary material

Table S1: Reference norovirus sequences for phylogenetic analysis

GenBank No.	Genotype	Region	Year
M87661	GI.1	US	1968
L07418	GI.2	GB	1991
U04469	GI.3	SA	1990
AB042808	GI.4	JP	1987
AJ277614	GI.5	GB	1989
AF093797	GI.6	DE	1997
AJ277609	GI.7	GB	1994
AF538679	GI.8	US	2001
HQ637267	GI.9	CA	2004
U07611	GII.1	US	1971
X81879	GII.2	GB	1994
U02030	GII.3	CA	1991
X76716	GII.4	GB	1993
AJ277607	GII.5	GB	1990
AJ277620	GII.6	GB	1990
AJ277608	GII.7	GB	1990
AF195848	GII.8	NL	1998
AY038599	GII.9	US	1997
AF427118	GII.10	DE	2000
AB074893	GII.11	JP	1997
AJ277618	GII.12	GB	1990
AY113106	GII.13	US	1998
AY130761	GII.14	US	1999
AY502010	GII.16	US	1999
AY502009	GII.17	US	2002
AY823304	GII.18	US	2003
AY823306	GII.19	US	2003
EU373815	GII.20	DE	2002
AY675554	GII.21	IQ	2002
AB083780	GII.22	JP	2003
KT290889	GII.23	PE	2010
KY225989	GII.24	PE	2013
GQ856469	GII.25	CN	2007
KU306738	GII.26	NI	2005
MG495077	GII.27	PE	2012
MG495079	GII.NA1	PE	2013
MG706448	GII.NA2	PE	2008
AJ011099	GIII.1	DE	1980
AF097917	GIII.2	GB	1976
EU193658	GIII.3	NZ	2007
AF195847	GIV.1	NL	1998
EF450827	GIV.2	IT	2006
AY228235	GV.1	US	2002
JX486101	GV.2	HK	2011
FJ875027	GVI.1	IT	2007
GQ443611	GVI.2	PT	2007
FJ692500	GVII.1	HK	2007
AJ844470	GVIII.1	JP	2004
AY130762	GIX.1	US	1999
KJ790198	GX.1	CN	2010
KP987888	GNA1.1	NL	2012
MG572715	GNA2.1	HK	2008

Table S2: Number of sequences detected per year

Year	Number of sequences
1980	1
2002	5
2004	35
2005	118
2006	138
2007	127
2008	197
2009	226
2010	313
2011	371
2012	144
2013	457
2014	752
2015	1989
2016	987
2017	804
2018	723
2019	239
2020	47

Table S3: Dynamic temporal change of human norovirus genotypes

Genotype	Year (Percentage)																		
	1980	2002	2004	2005	2006	2007	2008	2009	2010	2011	2012	2013	2014	2015	2016	2017	2018	2019	2020
GI.1	—	—	—	—	—	—	—	—	0.96%	0.81%	—	—	1.06%	0.05%	0.30%	0.12%	0.28%	—	—
GI.2	—	—	—	—	42.75%	—	—	—	—	—	—	0.22%	3.59%	—	1.22%	—	1.24%	—	—
GI.3	—	—	—	—	—	—	—	12.39%	—	2.70%	4.86%	0.22%	0.66%	0.15%	2.33%	—	0.83%	—	2.13%
GI.4	—	80.00%	—	—	1.45%	1.57%	—	—	0.96%	0.81%	3.47%	—	0.27%	0.05%	0.51%	—	0.41%	0.84%	—
GI.5	—	—	—	—	—	—	—	—	—	0.27%	—	—	1.99%	0.10%	0.81%	0.75%	1.24%	—	—
GI.6	—	—	—	—	—	0.79%	0.51%	—	0.64%	0.27%	—	0.22%	—	0.30%	5.27%	0.62%	2.07%	0.84%	—
GI.7	—	—	—	—	—	—	—	—	—	—	—	—	—	—	0.71%	—	0.28%	—	—
GI.8	—	—	—	—	—	0.79%	—	—	—	0.27%	—	—	0.80%	—	0.30%	—	—	—	—
GI.9	—	—	—	—	—	—	—	—	—	0.27%	3.47%	—	0.27%	—	0.51%	—	—	—	—
GII.1	—	—	2.88%	—	—	—	—	—	0.64%	0.81%	—	—	—	0.20%	—	1.00%	0.28%	—	—
GII.2	—		—	—	—		—	0.89%	1.92%	3.23%	—	—	2.13%	0.45%	13.07%	56.72%	17.15%	10.88%	85.11%
GII.3	—	—	34.29%	28.81%	2.17%	11.81%	5.58%	25.22%	13.42%	3.50%	31.25%	8.32%	21.01%	5.58%	7.40%	9.58%	16.32%	5.44%	2.13%
GII.4	—	20.00%	60.00%	64.41%	52.90%	65.35%	47.21%	28.32%	75.72%	78.71%	50.69%	38.95%	35.90%	42.08%	18.14%	8.08%	47.30%	75.31%	6.38%
GII.5	—	—	—	—	—	—	—	—	—	0.54%	0.69%	—	—	0.10%	0.51%	—	—	—	—
GII.6	—	—	—	—	—	—	—	7.08%	3.19%	3.77%	1.39%	14.88%	3.72%	1.26%	1.01%	1.62%	2.49%	2.93%	2.13%
GII.7	—	—	—	5.93%	—	—	—	0.44%	1.60%	0.27%	—	—	0.13%	0.15%	—	—	—	—	—
GII.8	—	—	—	—	—	—	—	—	—		—	—	—	0.10%	2.84%	—	0.55%	—	—
GII.12	—	—	2.86%	—	—	—	35.53%	24.34%	0.32%	1.35%	0.69%	0.88%	0.13%	0.25%	—	—	—	—	—
GII.13	—	—	—	—	—	—	0.51%	0.44%	0.64%	1.35%	3.47%	34.35%	1.33%	1.66%	6.79%	—	0.83%	0.84%	2.13%
GII.14	—	—	—	—	0.72%	—	0.51%	0.89%	—	0.81%	—	—	—	0.65%	0.10%	0.37%	—	—	—
GII.16	—	—	—	—	—	—	0.51%	—	—	—	—	—	—	—	—	—	—	—	—
GII.17	—	—	—	—	—	1.57%	—	—	—	—	—	1.97%	26.99%	42.53%	12.87%	13.93%	8.02%	2.93%	—
GII.20	—	—	—	0.85%	—	—	—	—	—	—	—	—	—	—	—	—	—	—	—
GII.21	—	—	—	—	—	18.11%	9.64%	—	—	—	—	—	—	4.32%	25.33%	6.84%	—	—	—
GIV.1	—	—	—	—	—	—	—	—	—	0.27%	—	—	—	—	—	—	—	—	—
GIX.1	—	—	—	—	—	—	—	—	—	—	—	—	—	—	—	0.37%	0.69%	—	—
